# Pleural Effusions: Their Diagnosis and Treatment

**Published:** 1893-03

**Authors:** Alfred Sheen

**Affiliations:** Senior Surgeon, Cardiff Infirmary; and Visiting Medical Officer, Cardiff Union Hospital


### PLEURAL EFFUSIONS: THEIR DIAGNOSIS AND TREATMENT.

BY

Alfred Sheen, M.D. St. And.,

*Senior Surgeon, Cardiff Infirmary; and Visiting Medical Officer, Cardiff Union Hospital.*

---

In order to deal with a pleuritic effusion, it is obvious that we must be sure that it exists, and this is not always so easy as at first sight it seems to be, unless great care is taken. We have to diagnose the presence of fluid, and then whether it is simply serous or purulent. The usual signs of fluid in the pleura are familiar enough, and where the quantity of fluid is large these signs are sufficiently obvious. In some cases, however, as Mr. Jacobson[1] points out, there may be other causes for some of the signs: for example, a lung partly solidified, and adherent with very thickened pleura, may render the breath sounds feeble or absent; bronchial breathing is often heard over effusions, especially in children; tactile vibrations may be present when strong adhesions run between the lung and chest wall, and when the lung is not extremely compressed; enlargement of the chest is only seen in large effusions and in young children.

Dr. Churton, of Leeds, has expressed the opinion that generally those affected with empyema lie (if on either) on the sound side, and those suffering from simple effusion lie on the affected side. Why this should be I do not know, and it does not accord with my experience. He also says that "fluid may be diagnosed pus, before aspiration, not so much by the condition of the patient as by the cause of the pleurisy."

Prof. Bacelli says that a thin homogeneous fluid, such as simple serous effusion, conducts the breath and voice sounds more intensely than an empyema. And Dr. Barlow states that "if a child be seen with general pallor and finger-clubbing, one ought to think of empyema rather than of the other causes of

[1] *Brit. Med. Journ.*, 1883, vol. i., p. 608.

clubbing; viz., chronic bone disease, bronchiectasis, and congenital heart disease."

Dr. Vincent Harris says that "effusions into the pleural cavity, even in large amounts, are not by any means always diagnosed — 1st, because the physical signs do not present themselves in orthodox form; 2nd, because it not infrequently happens that patients suffering from pleural effusion are so little inconvenienced that they . . . consult a medical man for symptoms, it may be, of dyspepsia; 3rd, because pleural effusion is very often secondary to some other affection within the chest . . . to which one's attention is more particularly directed."[1] Speaking generally, I decline to admit of such excuses for not finding fluid in the chest—each one of them would show, in any case, that a sufficiently careful examination of the patient had not been made.

Now, all this talk about the diagnosis of a pleural effusion, however interesting it may be from one point of view, seems to me to be superfluous; quite as superfluous as detailing a number of different methods of testing for sugar in the urine when you have one certain and efficient method. Surely we have one certain and all-sufficient method of diagnosing fluid in the chest, whether it be serum, blood, or pus, and that is the use of the hypodermic needle. An official of a certain town was blamed for not firing a salute on some occasion, and was asked his reason: he said there were nineteen reasons, the first being that they had no cannon; it was not considered necessary to give the other eighteen, of course. So here, there is one method of ascertaining the presence of fluid in the chest—the hypodermic needle—why trouble ourselves about the other methods?

One caution is necessary in the use of the needle for the purpose of diagnosis: it should be at least three inches long, and of sufficient strength to prevent its bending; I know of instances where failure has been the result of using an ordinary needle. A needle and syringe similar to the one used for the injection of ether seems to me to answer all requirements.

Fluid being found, what are we to do? Leave it to nature? This used to be the routine practice, but it is bad practice in

[1] *Brit. Med. Journ.*, 1890. vol. i., p. 1061.

many cases of serous effusion, and in all cases where there is pus. What is to be the treatment of serous effusions? It is said to be a fact that in some cases such an effusion, if left to itself, disappears in a comparatively short time. "These effusions are," says Mr. Jacobson, "not like ascites, requiring mechanical aid; they are more or less inflammatory and tend to be absorbed;"[1] and he recommends the trial of the iodides of potassium and iron, a dry nutritious diet, and the occasional use of laxatives. Such treatment may be useful in some cases, as where the fluid is small in quantity, but where it is otherwise I think that time is lost, and much risk may be incurred thereby. What have we to deal with? An effusion into a cavity with a somewhat rigid outside wall, a compressible lung inside, and an inflamed pleura with little or no absorbing power because inflamed. The old dread of wounding the peritoneum seems still to cling to us when dealing with the pleura. My own feeling is in favour of removing serous effusion by aspiration, under antiseptic precautions, unless the fluid is small in amount. I generally use the bottle aspirator, or Dieulafoy's—the former by preference, as all the actions in using it are steadier. The site of puncture should be posteriorly and low down, and the patient should be in the recumbent posture. Dr. Bowditch's rule is—find the inferior limit of the sound lung behind, and tap two inches higher than this on the side of the effusion in a line perpendicular from the inferior angle of the scapula.

Mr. Jacobson speaks of the dangers of leaving the effusion, and of the risks of paracentesis. Of the former he mentions—risk of sudden death in large quiet effusions, specially on the left side, where the heart may be displaced, causing twisting of the inferior vena cava; risk of lung being more and more tied down by adhesions; risk of tuberculosis, and of purulent transformation. These are all dangers more or less. Of the dangers of paracentesis he mentions shock, syncope, embolism (to be prevented by maintaining the horizontal position and drawing off the fluid slowly), and œdema of lungs, the symptoms of which are, that shortly after tapping (usually large effusions)

[1] *Loc. cit.*

urgent dyspnœa comes on, with frothy serous expectoration rich in albumen; and death in 24 to 48 hours. Personally, I have been fortunate enough not to meet with any of these dangers.

How long shall we leave a serous effusion in the chest without tapping it? Of course, we must bear in mind the dangers of leaving it, especially if it be large in amount. Dr. V. Harris says, if the serum is small in amount we may wait. He has no faith in drug treatment, but thinks he has seen good results from blisters and iodide of potassium in certain cases. He advises generous diet, cod-liver oil, iron, &c., in any case, with the view of improving the general health, and that the patient should get up part of the day, and that, if the weather be fine and warm, he should have moderate exercise in the open air. If, in the course of a week, there is evidence of diminution of the fluid, it may be left—not more than two weeks if no diminution—and, if increasing, he advises tapping at once. In children, he says, there is a strong tendency to the rapid spontaneous absorption of serum, and if tapped the operation is peculiarly successful. The effusion in them is, however, frequently purulent.

Are all large effusions to be tapped? My friend Dr. D. R. Paterson tells me that he once saw a lady going downhill rapidly from extensive disease of the left lung, when a large effusion took place into the left pleura, and the lung disease was apparently arrested; at any rate she rapidly increased in weight and improved immensely in her general health. He asks: "Was not the effusion here beneficial?" I should imagine it was so.

Sometimes a serous effusion has rapidly become purulent after tapping. In such a case I should suspect want of care and cleanliness in the use of the aspirator, unless there were some other possible cause, such as a broken-down constitution.

Cases are on record of rapid transition from serum to pus, as proved by the hypodermic needle. Much discussion has recently taken place as to the frequency with which pneumonia is the precursor of empyema, some writers going so far as to claim all cases as secondary to pneumonia. Ziemssen not long ago stated that during the last four years he met with over twenty such cases. Dr. D. R. Paterson tells me of a case he

3

saw in consultation—a gentleman, about 32 years of age, who was, three weeks before, seized with acute pneumonia of the left base, all the physical signs being present, as well as rusty sputum. The temperature fell about the tenth day, but rose again, going up at night to 101° and 102°. There were few signs pointing to anything other than a consolidated lung, beyond an absence of the bronchial breathing just above the lower limit of the lung. The needle showed pus, and the chest was incised, evacuating about a pint of thick creamy pus. The temperature fell at once, and, with the exception of a transient rise due to blocking of the drainage tube, the patient was well in three weeks, and all discharge had ceased.

Having ascertained the fluid in the chest to be *pus*, what is to be done, and what points have to be considered? I think I am correct in saying that all surgeons, or almost all, are now agreed that the only proper treatment is free incision at the earliest possible moment; and this is only reasonable treatment, for have we not an abscess to deal with, and do we not advocate this treatment in all other abscesses? We have been slow in arriving at this conclusion, and the only reason which occurs to my mind is that dread, which I have spoken of before, of opening serous cavities. The introduction of what is called "antiseptic treatment" has undoubtedly been a large factor in disposing of this fear, but free incision is to me as old as my student days. My old teacher, in 1858–59, a very astute and learned Scotchman, gifted with a large measure of common sense, was wont to tell us in his lectures that the only satisfactory treatment of an empyema was to make a free incision into the pleural cavity so that the air could rush in and out; and this was long before the advent of antiseptic treatment.

At the International Congress in 1881, there was introduced by Dr. C. Gerhardt, of Würzburg, a discussion on the surgical treatment of empyema in children.[1] With regard to a small empyema, he was of opinion that it might be cured spontaneously by absorption (I doubt it); that a single aspiration sometimes resulted in a complete cure, and that another favourable termination was by expectoration. After all, he advocates the

[1] *Brit. Med. Journ.*, 1881, vol. ii,, p. 523.

free opening of the chest under antiseptic precautions, and is of opinion that washing out the pleura is not free from danger. Mr. Richardson Cross (Bristol) advocated the early removal of the fluid to ensure the expansion of the lung, and advised an incision in the eighth or ninth intercostal space, with antiseptic precautions, *if* aspiration failed after two trials. I think if my esteemed friend had continued to devote his attention to general surgery, he would long ago have omitted the "two trials." Mr. R. W. Parker believed that aspiration in childhood, two or three times, was the best treatment, and ought always to be adopted before other measures were tried. In adults he advocated the injection of filtered and carbolized air into the pleura, stating that it helped to replace the fluid, lessened the dragging sensation often felt, and prevented re-accumulation. Surely the lung is the proper substance to replace the fluid.

I find my friend, Dr. Ward Cousins, saying: "During the last few years free incision in empyema has been extensively and successfully employed, although many surgeons still regard it as a radical measure."[1] He has his own little instrument for the operation of incision, but, after all, the simplest instruments are the best, and in this instance I prefer an ordinary scalpel.

Dr. Eddison, of Leeds, says, in 1883, "There is still a great deal of unnecessary hesitation about incising the chest, even when an empyema is known to exist."[2] He lays stress on the importance of free drainage. One has seen over and over again the temperature going up and the progress of the case retarded, owing to interference of the free exit of pus from blocking of the drainage tube. This is only part of the first principles of surgery now-a-days.

Dr. Rickards, of Birmingham, in reporting ten consecutive hospital cases,[3] says that "given a case of empyema, I think we ought, at the earliest possible opportunity, to incise the chest and let out the pus," and advocates the use of the aspirator first, to ensure the spot at which the incision is to be made. Surely he would not say "I think" in dealing with an abscess in any other situation.

[1] *Brit. Med. Journ.*, 1883, vol. ii., p. 112. [2] *Ibid.*, p. 618.

[3] *Ibid.*, 1886, vol. ii., p. 969.

At a discussion at the Medico-Chirurgical Society in Edinburgh, in 1887,[1] aspiration was still spoken of as a method of treatment, but free drainage, without excision of rib was advocated, making a double opening and using a continuous drainage tube. The opening was not to be too low down, or, during subsequent retraction, proper drainage would be interfered with by elevation of the diaphragm. The diagnosis of pus in the pleura was stated to be difficult by one speaker; and another advocated repeated tapping, at intervals of two or three days, between the fifth and sixth ribs. Another speaker objected to the routine use of the subcutaneous syringe; and Dr. Bramwell spoke of the difficulties of diagnosis. I would humbly submit that the diagnosis is simple enough in almost all cases if a proper syringe is used.

Mr. Wheelhouse, of Leeds, recommends[2] the space between the eighth and ninth ribs (eighth interspace) for aspiration or knife, and he further advises that if, on introducing the finger, it is found that an opening lower down would facilitate emptying, we should not hesitate to make it, as the other will heal up rapidly under antiseptic precautions.

Mr. Norman Porritt, in his prize essay on *The Operative Treatment of Intra-thoracic Effusion* (1883), gives the following directions: "Place the arm and elbow at the side, and mark the spot at which the lower angle of the scapula rests upon the chest; then place the forearm over the head, and again mark the spot on which the lower angle now rests; draw a line from one of these marks to the other; and then, from the centre of that line, drop another perpendicularly down for an inch, and its end will fall on the eighth intercostal space, where the interval between the eighth and ninth ribs is the widest, and the parts are least covered by muscles."

Dr. Vincent Harris, writing in 1890, advocates free incision and irrigation with a large quantity of antiseptic fluid. He does not, however, condemn the treatment of repeated tapping; he says: "Of course there is no actual reason why the 'close' method should not be tried first of all; it may answer sometimes."[3] If regular and prolonged washing out does not suc-

[1] *Brit. Med. Journ*, 1887, vol. i., p. 460. [2] *Ibid.*, vol. ii., p. 1141. [3] *Loc. cit.*

ceed, and if the health is giving way, suggesting the possibility of amyloid disease, he recommends resection of fifth and sixth ribs, done in axillary line, and one or two inches removed. I presume he is here referring to chronic cases. He emphasises two details in the post-operative treatment:

1. The necessity of getting the patient out of bed as soon as possible, and into the open air without delay, by which, he asserts, the period of convalescence is cut short and the discharge diminished.

2. As soon as possible—early in the "close" operation, and as soon as the discharge is diminished to half-an-ounce a day in the open operation—to make the patient expand the chest by regular, frequent, but moderate dumb-bell exercise, or by drilling. He states that he has had the best results where this has been done.

Having a case of empyema, I should incise it freely at the earliest possible moment. The operation may best be considered under the following headings:

*Position of incision.*—I should select the eighth interspace behind the mid-axillary line, but I believe with Dr. Eddison that the particular point at which the chest is incised is not of much importance. I use an ordinary scalpel, and plunge it straight in at the point selected, keeping close to the upper surface of the rib to avoid wounding the intercostal artery, being careful for nothing except to make a free incision. The patient is in the recumbent position, lying somewhat on the sound side, and an anæsthetic has been given. In the most favourable cases, as the pus flows out, the lung expands.

*Antiseptic precautions.*—I have given up using the spray. The part to be operated on is thoroughly cleansed with soap and water, and then with a 1 to 20 carbolic solution, and a compress of 1 to 40 carbolic kept on up to the time of operation. The instruments are boiled in water, and then placed in a dish containing carbolic 1 to 40. The hands are thoroughly cleansed with soap, brush, and hot water, and then with carbolic 1 to 20.

*Drainage tubes.*—I use one or two ordinary red rubber tubes, just long enough to enter the pleural cavity, and secure them with safety pins. One cannot help being amused at seeing, from time to time, the various ingenious inventions in the way of drainage tubes for empyema.

*Syringing out of cavity.*—I have never done this. I think, however, if done once, at the time of operating, it will facilitate removal of lymph, a warm solution of carbolic acid 1 in 80 or 100 being used. I would use an ordinary Higginson's syringe, washing out very gently. I strongly deprecate the repeated washing out of the pleural cavity.

*Excision of ribs.*—I have never had to excise a rib; and Mr. Wheelhouse, of Leeds, who has had a much larger experience than I have, has "never been driven to this expedient."

Rudolph states[1] that it is the practice at the Magdeburg Hospital to resect a rib and to wash out the pleural cavity in all cases of empyema; but Herz maintains, in reply to Rudolph, that "except in some unusual cases, resection of a rib complicates the operation as washing out the pleural cavity does the after-treatment."[2]

In the discussion which took place on a paper read by Mr. Godlee at the Nottingham Meeting of the British Medical Association, last July,[3] the consensus of opinion was against excision, except in some few cases. Mr. Godlee, in his opening paper, advocated "removing a piece of rib as a routine practice with very few exceptions." He says: "1. It allows of the best possible exploration of the pleura with fingers and probes. 2. It permits of the evacuation of masses of lymph. 3. It obviates, to a great extent, the difficulty, which is common afterwards, of retaining or reintroducing the tube." I am not convinced by these statements. I venture to think that the less we meddle with "fingers and probes" the better, and that if a sufficiently free incision—from two to three inches—is made, there will be no trouble with masses of lymph or retention and reintroduction of tube.

*Dressings.*—Having opened the chest, let out the pus, and inserted drainage tube, I usually dust some iodoform powder about the wound, place a piece of green protective over the tube, and apply a thick pad of sublimate wood wool, securing

[1] *Brit. Med. Journ.*, 1892, vol. ii., Epitome, p. 85. [2] *Ibid*,, p. 81.
[3] *Ibid.*, vol. ii., p. 829.

this by a bandage, and dressing again when the discharge comes through. If the temperature goes up, it is usually an indication of retention from blocking of drainage tube, in which case I remove it, cleanse it, and replace it.

*Anæsthetics.*—There seems to be some difference of opinion as to the use of these. Speaking generally, I think it is an advantage in any operation to have the patient unconscious, as one never knows beforehand what will have to be done, and how long the operation and the subsequent proceedings will last. If the patient objected to an anæsthetic, or if there was any other reason for not giving it, I would operate without it, or inject ¾ grain of cocaine at the seat of incision. Mr. Wheelhouse pleads for the use of ether "where the operation is likely to be prolonged, or to cause much pain," and has never seen any harm from it. Otherwise he prefers to operate without it, as a conscious patient can afford much help by change of position, greater or less efforts of expansion and contraction of chest walls, and otherwise. In all the cases I have had to deal with, an anæsthetic has been given.

Of tuberculous empyema I have had no experience; but reasoning from analogy I fail to understand why it should not be treated as vigorously as any other tuberculous abscess in an accessible situation.

I now give seven briefly recorded cases which may be taken as illustrative of various forms of pleuritic effusion:

CASE I.—**Aortic Disease; effects of backward pressure; successive aspirations; death.**

John K., æt. 45, stonemason, admitted to hospital on August 27, 1890.

*History.*—In February had influenza, pain in right breast and shortness of breath; this lasted a fortnight, but the patient never got rid of his short breath. He worked up to the beginning of June, and then went "on strike." The breathing became more embarrassed, cough came on, and he had "asthmatic" attacks. Previously healthy. Never had rheumatism. Has worked hard, lifting heavy stones. Has lost weight recently.

Sept. 3. *Present condition.*—Well built; shortness of breath has specially increased the last three days; cannot lie down for long; no pain; some cough; no expectoration, jaundice, or cyanosis; œdema of ankles. *Heart:* apex beat in sixth left interspace, pulsating as far as anterior axillary line; loud double aortic murmur at second left intercostal space, propagated down sternum; collapsing pulse. *Liver:* lower border on a level with umbilicus; tender. *Spleen:* palpable just below ribs. *Lungs:* right side dull in front from first interspace downwards, and behind from just above lower angle of scapula; breath-sounds feeble; modified ægophony; serous fluid by hypodermic needle.

Oct. 10. Increase of distress in breathing, and in swelling of legs, ankles, and abdomen. Aspirated at right base behind, and 68 oz. of serum removed. Patient relieved, able to lie down and sleep.

Oct. 22. Fluid re-accumulated. Aspirated, and 80 oz. removed.

Nov. 1. Aspirated. 88 oz. removed.
,, 8. ,, 100 ,, ,,
,, 12. ,, 76 ,, ,,
,, 17. ,, 72 ,, ,,
,, 20. ,, 76 ,, ,,
,, 22. Patient relieved after each tapping, and slept well the same night. Swelling of legs nearly all gone; less in abdomen. Breathing much easier.

Nov. 25. Aspirated. 74 oz. removed.
,, 29. ,, 72 ,, ,,

Dec. 3. Hardly any fluid in chest. Patient feels much better. Pulse regular. Swelling in legs gone.

Dec. 9. Aspirated. 72 oz. removed.
,, 13. ,, 86 ,, ,, Pulse 96, regular. Cardiac dulness increased from mid-sternal to mid-axillary line, ending in liver dulness below and in second intercostal space above. Sounds as before. Epigastric pulsation.

Dec. 31. Aspirated. 4 oz. removed.

Jan. 5, 1891. Shortness of breath came on; fluid in pleura not increased; loud friction sound heard over sternum, synchronous with heart beat, and not affected when patient stopped breathing.

Jan. 6. Patient worse. Cardiac dulness increased; friction sound not heard; heart sounds distant and indistinct. Patient gradually sank, and died at 6 a.m. on January 7.

*Necropsy.*—Large, hypertrophied, much dilated heart; aortic valves much diseased; aorta atheromatous; heart surface and pericardium covered with lymph and a large quantity of fluid in pericardial sac.

CASE II.—**Pleurisy, with effusion; aspiration; recovery.** (From notes by Dr. D. R. Paterson.)

William R., æt. 48, labourer, admitted to hospital on August 6, 1890.

*History.*—About one week before admission taken with shortness of breath and pain at epigastrium; shortness of breath on exertion; slight cough; able to lie down; no œdema of feet. A year ago was in hospital with what appeared to be kidney trouble: swelling of legs and dark-coloured urine. Since then has been well until this attack came on suddenly.

*Present condition*.—Has some dyspnœa, but breathing somewhat long drawn, suggesting renal trouble; pain at end of sternum. No œdema, cyanosis or jaundice. Lies down comfortably. Pulse, 108; respiration, 36; temperature, 99.2°. Cough troubles him; expectorates a little mucus, never rust-coloured. *Heart*: apex beat fifth left interspace inside nipple; sounds normal. *Lungs:* right side larger than left; dulness at right base behind and in axilla, and in front from third rib down. Breath-sounds not absent behind, but vocal resonance and vocal fremitus diminished. Fluid, clear serum, with hypodermic needle. Urine high-coloured, almost smoky; small amount of albumen. Bowels open; enuresis at night. Takes ammon. carb. gr. iij., pot. bromid. gr. xv., tr. camph. co. m. xv., every 4 hours in water.

On the 27th he had been up for a week, feeling well. Dulness cleared up very considerably behind, and there was more expansion of chest. No albumen in urine. Discharged next day.

Sept. 10. Re-admitted. Only worked a week and was obliged to give up owing to shortness of breath and pain between the shoulders. Has got thinner. Chest not so prominent on right side as formerly. Dulness posteriorly in left scapular region; impairment at left apex, with increased vocal resonance and a few moist râles. At right base respiration is harsh. *Heart:* apex in fifth left interspace internal to nipple; first sound reduplicated. Urine free from albumen. No rise of temperature at night. Put on pot. iod. gr. x., liq. hydrarg. perchlor. ʒj., t.d. ex aq., on Oct. 6.

Oct. 26. Much better. Very little cough; note more resonant over left scapular region, and no *râles* at left apex. Moist sounds on deep inspiration at right base, but note fairly clear. Pigmented spots and their arrangement along intercostal spaces suggested syphilis.

Dec. 10. Discharged much improved.

Case III.—**Pleurisy, with effusion; aspiration; recovery.**

Frederic K., æt. 48, cooper, admitted to hospital on December 26, 1890.

*History*.—Five weeks ago taken with shivering and pain in right side, and laid up. Pain continued off and on, and breath became shorter; slight cough. One week ago breathing became worse, and he went to a doctor, who said he had inflammation of the lungs and pleurisy, and painted his chest twice with tincture of iodine, which made the skin very sore. Two days before admission had to be propped up in bed. Was always strong and healthy before this illness.

*Present condition.*—Appears very ill. Breathing laboured and difficult; no movement on right side of chest; vocal fremitus absent and vocal resonance diminished; no respiratory sounds; ægophony. Heart slightly displaced to left; pulse 85, regular; Heart-sounds normal. Needle introduced, and clear fluid drawn off. Aspirated at once, and 72 oz. of serous fluid removed.

Dec. 27. Slept well last night, and feels comfortable. Perspired freely for the first time for six weeks. Can lie on either side; before tapping was obliged to lie on right side or back. No distress, œdema, cyanosis or jaundice. Urine free from albumen.

Dec. 30. Comfortable; no distress in breathing; dulness not increased.

Jan. 6, 1891. Comfortable; more resonance in front, also behind as far down as spine of scapula. 80 oz. of clear serum removed by aspirator. He continued to improve, and was discharged at his own request on February 14, feeling perfectly well, but with some dulness remaining at extreme base.

CASE IV.—**Pleurisy; empyema; incision; recovery.**

Peter V., æt. 24, groom, admitted to hospital on May 14, 1884.

*History.*—Illness began about eight weeks ago with a pain in the left side like a stitch; continued his work for a fortnight, when he went to bed and was attended by a doctor. His chest had been aspirated five times, giving exit to serous fluid. Ten days before admission he had rigors, with a rise of temperature.

*On admission.*—Left chest $\frac{3}{4}$ in. larger than right; intercostal spaces obliterated; movements deficient; vocal fremitus absent except at apex, deficiency of resonance; bronchial sounds very feeble; ægophony. Heart beats to right of sternum, impulse being most felt to inner side of right nipple. Troublesome cough, expectoration scanty. Pulse, 80; temp., 102.2°.

May 16. Opened antiseptically by incision behind mid-axillary line in eighth interspace, 26 oz. of pus discharged; dressed antiseptically with salicylic silk and carbolic gauze, an india-rubber drainage tube having been inserted and secured by a pin. Heart resumed its normal position.

May 25. Bronchial sounds heard over front and back distinctly. On June 10 and 14 there was a rise of temperature due to obstruction of tube. On the 19th patient was up, looking and feeling better. On July 30 only about $\frac{1}{2}$ oz. of pus escaped after extraction of drainage tube. The dressings were renewed about every three or four days, and the temperature was practically normal, with occasional evening rises at infrequent intervals. The case was a long one, as most of these cases are, lasting thirty-six weeks from the commencement of the illness, and three years elapsing before the wound finally closed.

I saw this patient in February, 1889, when he was in good health and active work as a groom, and he has remained so ever since.

CASE V.—**Pleurisy; empyema; incision; recovery.**

J. S., æt. 17, clerk. First seen February 10, 1887. Had caught a "chill," and had a temperature of 101° F. 11th: pulse, 100; temperature, 103.6°. 12th: Pulse, 112; temperature, 104°. Briefly, this was a case of limited pneumonia at the right base, quickly followed by pleurisy with effusion, which progressed satisfactorily till Feb. 21, and which I hoped would end in resolution; the temperature went up, however, on the 22nd to 101.4°, and on the 23rd to 103°; the patient was evidently worse, and the breathing much distressed. The right side measured 18¼ in. and the left 16¾ in. On the 25th the right side was 18¾ in. Pulse, 112; temperature, 102°.

Feb. 26. Chloroform given. Aspirated right chest posteriorly with hypodermic needle, found pus, incised pleura under carbolic spray, and removed between three and four pints of pus; antiseptic dressings, which were changed in two hours. *Vesp.*: pulse, 90; temperature, 99°; a good deal of perspiration. A fair sized india-rubber drainage tube was inserted, secured by a nursery pin. The dressings were subsequently renewed about every other day. On March 4, the temperature went up to 105.4° from some unknown cause, coming down to normal next morning, and remaining so till the 10th, when it went up to 104°, probably from inefficient drainage and consequent retention of pus. This case lasted from nine to ten weeks, and ended satisfactorily. There was good expansion of the lung, and the patient soon after his recovery went to fill a situation in South America, and the latest reports about him are that he is as well as ever he was.

CASE VI.—**Empyema following pneumonia; incision; recovery.** (From notes by Dr. D. R. Paterson.)

Charles R., æt. 26, fireman, admitted to hospital on May 31, 1892. Was taken ill with vomiting and shivering one month ago. Two days before, a case of scarlet fever occurred in same house in which the patient was. Was quite well previous to attack, and had no cough. Went to bed; cough came on, and for first two days he spat up thick phlegm mixed with blood, and then frothy phlegm. Had severe pain in left chest. About seven days ago, feeling much better, was allowed to get up, and went out several times, but feet began to swell, breathing became embarrassed, and there was considerable sweating. Had been drinking a good deal previous to the attack; always previously healthy. No family history of phthisis. Temperature, 102.8°.

June 1. *Present condition.*—Well built; somewhat pale; has lost flesh; skin harsh. Propped up in bed, cannot lie down or on either side; cough severe occasionally, worse at night; sputum muco-purulent; slight œdema lower part of back, none in legs. Pulse, 116; respiration, 40, with slight distress; temperature, 101°. *Heart:* apex pushed to right side. Usual signs of effusion.

June 2. Chest opened last night in eighth interspace, in front of angle of scapula, and 70 oz. of thin pus let out. After getting into bed breathing became distressed; some collapse and cold sweat, which continued more or less all night. Now easier; breathing frequent and shallow; resonance and breath-sounds over upper part left lung. Pulse, 98; temperature, 99.6°.

June 3. Much easier. Chest washed out yesterday and to-day with warm boracic lotion. Pulse, 88; temperature, 101.2°. Some crepitation over front of upper left lobe.

June 15. Dressed daily; eats and sleeps well. Only a trace of discharge. Resonance over left chest, except at extreme base behind. Temperature normal since the 3rd.

June 23. Up. Tube removed; no discharge from wound. Expansion still much impaired.

July 6. More expansion; note deficient, and breathing weak below angle of scapula. *Heart:* apex below nipple. On deep inspiration some shooting pains in left shoulder. Has picked up flesh.

July 9. Feels quite well. Discharged.

CASE VII.—**Empyema following injuries; incision; recovery.**

Geo. G., æt. 35, ballastman. Admitted to hospital on Oct. 24, 1882, with incised wound on forehead, bruised shoulder and hip; and made out-patient on Nov. 1. Re-admitted on Nov. 11 with severe pain in back of head, bagging of pus under scalp; general malaise; temperature of 104.6°; some cough, and pain in chest.

Nov. 16. Respiratory movements defective on right side; vocal fremitus and resonance much diminished to lower angle of scapula; ægophony well marked.

Nov. 23. Dulness now extends to supra-spinous fossa behind and supra-clavicular fossa in front.

Nov. 30. Tapped with an ordinary trocar and cannula by the house-surgeon, and 136 oz. of pus escaped. Resonance was then equal on both sides, except at right base.

Dec. 14. Condition of patient not much improved, the chest having rapidly filled again. I made a free incision into the chest in a dependent position in mid-axillary line, under chloroform, with carbolic spray and all the usual antiseptic precautions. Drainage tube inserted. Next day right chest resonant all over.

On Jan. 8, 1883, the patient was put on cod-liver oil, and the case went on very satisfactorily until

Feb. 12, when, as it was a warm sunny day, he went out of doors for the first time.

On the 14th his temperature went up, and remained up till the 22nd, when there was a discharge of 16 oz. of pus. There was evidently not a sufficiently free exit (confinement of pus caused rise of temperature). A larger drainage tube was inserted, and from this time onwards till his discharge, in the middle of April, he did well and rapidly improved in every way. I saw him in February, 1889, when he was in good health and able to do a hard day's work. There was good expansion of the lung, and the right side was only ½ in. smaller than the left.

